# Impact of Caffeine Intake Strategies on Heart Rate Variability during Post-Exercise Recovery: A Systematic Review and Meta-Analysis

**DOI:** 10.2174/011573403X289842240307114736

**Published:** 2024-03-14

**Authors:** Bianca Araujo Almeida, Anderson Pontes Morales, José Ricardo Claudino Ribeiro, Felipe Sampaio-Jorge, Yasmin Garcia Ribeiro, Thiago Barth, Beatriz Gonçalves Ribeiro

**Affiliations:** 1 Research and Innovation Laboratory in Sports and Nutrition Sciences (LAPICEN), Federal University of Rio de Janeiro (UFRJ), Macaé, RJ, Brazil;; 2 Postgraduate Program in Bioactive Products and Biosciences, Federal University of Rio de Janeiro (UFRJ), Macaé, RJ, Brazil;; 3 Laboratory of Natural Products, Federal University of Rio de Janeiro (UFRJ), Macaé, RJ, Brazil;; 4 Macaé Sports Secretary, City Government of Macaé (PMM), Macaé, RJ, Brazil;; 5 Laboratory of Applied Physiology for Health, Performance, and Physical Education (LAPHPE), Higher Institutes of Education of CENSA (ISECENSA), Campos dos Goytacazes, RJ, Brazil;; 6 Postgraduate Program in Nutrition, Institute of Nutrition, Federal University of Rio de Janeiro (UFRJ), Rio de Janeiro, Brazil;; 7 Minas Tennis Club, State University of Minas Gerais, Minas Gerais, Brazil

**Keywords:** Dietary supplements, caffeine, metabolism, heart rate, autonomic nervous system, exercise

## Abstract

**Objectives:**

The objective of this systematic review and meta-analysis is to evaluate the influence of caffeine (CAF) intake strategies, taking into account their form, timing, and dosage, on heart rate variability (HRV) indices in the post-exercise recovery period.

**Methods:**

The meta-analysis adhered to the Preferred Reporting Items for Systematic Review and Meta-Analysis (PRISMA) guidelines and is registered in the PROSPERO database (CRD42023425885). A comprehensive literature search was carried out across MEDLINE, Web of Science, LILACS, and SCOPUS, concluding in May 2023. We concentrated on randomized clinical trials comparing CAF supplementation effects to placebo on HRV indices post-exercise in active adults aged 18 and above. The primary endpoint was the assessment of HRV indices, measured both prior to and following exercise.

**Results:**

Of the 10 studies included, 7 were used for the meta-analysis, and all contributed to the systematic review. The research explored a variety of CAF strategies, spanning different forms (capsule, drink, gum), times (10, 45, 60 min) and doses (2.1 to 6.0 mg/kg). The outcomes revealed no substantial variations between the placebo and CAF conditions in terms of both the square root of the average of successive squared differences between adjacent RR intervals (RMSSD) (standardized mean difference (SMD) -0.03, 95% CI -0.265 to 0.197, *p=*0.77) and high frequency (HF) index (SMD -0.061, 95% CI -0.272 to 0.150, *p=*0.57). Furthermore, meta-regression analysis, employing a fixed-effects model and accounting for the administered CAF doses, revealed no significant correlation between caffeine doses and HRV indices (*p>*0.05).

**Conclusion:**

In conclusion, there is moderate-certainty evidence suggesting that different CAF intake strategies, encompassing aspects such as form, time, and dose, do not have a significant impact on HRV indices recovery post-exercise (*i.e.*, vagal modulation).

## 1 INTRODUCTION

Caffeine (CAF), recognized as 1,3,7-trimethylxanthine, is a supplement widely used by physically active individuals and trained athletes [1] due to its ergogenic impact on both endurance [2] and strength exercises [3-5]. Given its predominant use among athletes, it is essential to validate the evidence on safe caffeine intake considering physical exercise, particularly in the cardiovascular system.

Although moderate caffeine intake can provide advantages, such as heightened alertness and enhanced physical performance, it is of utmost importance to ensure careful monitoring of its intake due to its inotropic, tachycardic, bronchodilating, and gastric secretion-stimulating effects [6, 7], particularly in vulnerable individuals. Concerning habitual consumption, caffeine intake from all sources up to 400 mg per day (approximately 5.7 mg/kg of body weight per day for a 70 kg adult) throughout the day does not raise safety concerns for healthy and physically active individuals, according to the European Food Safety Authority [8, 9]. Additionally, an exclusive daily recommendation of 400 mg for athletes has been authorized, as established by the Brazilian National Health Surveillance Agency [10].

Specifically, the safety of CAF usage depends on strategies, such as dose [6], time [7], and form of ingestion [11] when employed for ergogenic purposes. Two suggested mechanisms for how CAF influences cardiovascular function include the activation of the sympathetic autonomic system through catecholamines released into the bloodstream [12] and the inhibition of adenosine receptors (A1, A2A, A2B) in the central nervous system [13]. CAF application targets heart cells and vessels as a stimulant to induce arrhythmias through Ryanodine receptors and their altered forms. By binding to the Ryanodine receptor, caffeine prompts calcium flows, potentially leading to arrhythmias associated with the positive inotropic effect of CAF [14]. Employing heart rate variability (HRV) as a non-invasive method to evaluate cardiac autonomic function holds potential for assessing the cardioprotective effects of exercise [15, 16]. However, CAF might influence this HRV index measurement [17]. Analyzing successive heartbeats (R-R intervals) enables an indirect evaluation of the autonomic nervous system's activity in the heart, encompassing both parasympathetic and sympathetic components. Consequently, if CAF causes a postponed reactivation of the parasympathetic nervous system post-exercise (vagal reactivation), it could be deemed an adverse cardiac result [12]. Earlier research [17, 18] investigated the effect of CAF on the autonomic nervous system through HRV. The findings suggest a decrease in HRV linked to dose [19], time [20, 21], and form of ingestion (such as drinks, gum, and capsules) [11, 21, 22]. A reduction in HRV may indicate potential adverse effects on the heart, increasing the risk of cardiac arrhythmias, such as atrial fibrillation and cardiovascular stress [23].

In a recent systematic review, the safety of CAF was assessed based on HRV recovery. However, this study had methodological limitations, including the range of years covered by the included studies, the absence of quantitative analysis, and the inclusion of studies that involved substances mixed with CAF. These limitations impeded the formulation of a conclusive and definitive assessment [24]. Another systematic review accompanied by a meta-analysis remained inconclusive, mainly due to certain limitations, including the lack of analyses related to the form, dosage, and timing of CAF ingestion prior to initiating exercise [25]. Consequently, a thorough examination of the safety implications surrounding CAF consumption by athletes and active individuals engaging in maximal exertion becomes a necessity. To address and potentially mitigate the limitations identified in previous studies [24, 25], the objective of this study is to perform a systematic review and meta-analysis to assess how CAF intake strategies, encompassing form, timing, and dosage, impact HRV indices during the post-exercise recovery period in healthy, physically active adults, and athletes.

## 2 MATERIALS AND METHODS

### 2.1 Registration

The review adhered to the guidelines outlined by the Preferred Reporting Items for Systematic Reviews and Meta-Analyses [26] and has been registered in the PROSPERO database (CRD42023425885).

### 2.2 Search Strategy and Study Selection

The search strategy and study selection involved utilizing four databases, namely MEDLINE (*via* PubMed), Web of Science, Latin American and Caribbean Health Sciences Literature - LILACS and SCOPUS), utilizing the keywords “Autonomic Nervous System” OR “Heart Rate Variability” AND “Caffeine” AND “Exercise” (Table **1**), we identified relevant studies. Subsequently, the selected articles were imported into the Rayyan QCRI program (Qatar Computing Research Institute, Qatar) to eliminate duplicate entries identified during the search. The screening process in the Rayyan program involved applying filters such as “study in humans” and “Randomized Controlled Trials,” followed by further screening based on reading the title and abstract. The eligibility stage, representing the ultimate phase for article inclusion, was conducted through a meticulous reading of the articles by two independent reviewers (APM and FSJ). In the event of a disagreement at this stage, an additional reviewer (BGR) was consulted to provide a resolution.

### 2.3 Eligibility Criteria

The inclusion criteria, following the Population, Interventions, Comparisons, and Outcomes (PICOS) framework, were defined as follows:

Population (P): Individuals aged 18 and above, in good health, physically active, and trained.

Intervention (I): Caffeine.

Comparison (C): Placebo, including dextrose capsule, opaque gelatin, starch, maltodextrin or pulp, coffee without caffeine, bottled mineral water with artificial refreshment, and magnesium silicate.

Outcome (O): Measurement of HRV indices both before and after exercise.

Study Design (S): Randomized clinical trials (RCTs).

### 2.4 Data Extraction

The process of data extraction involved providing details on the author, study design, characteristics of study participants, intervention, and the results of each respective study. In cases where inconsistencies were identified, these were addressed through discussion, and any missing data were sought by reaching out to the respective study authors. In instances where no response was received from the authors, the Web Plot Digitizer was utilized to extract data from charts. This phase was concluded independently by two reviewers (APM and FSJ) within the research group.

### 2.5 Assessment of Risk of Bias and Study Quality

Two reviewers, APM and FSJ, independently conducted the risk of bias assessment for the included trials. Any disagreements were resolved through discussion with a third reviewer until a consensus was reached. The critical appraisal of each study was based on a revised version of the Cochrane risk of bias tool (RoB2) [27] and its supplement [28]. A summary assessment of bias was generated using an Excel tool designed to implement the RoB2 [28]. The bias assessment approach focused on adhering to the intervention described in the trial protocol, known as the 'per-protocol' effect.

### 2.6 Evidence-level Assessment

The certainty of the evidence was independently evaluated by two authors, utilizing the Grading of Recommendations Assessment, Development and Evaluation (GRADE) methodology as per Atkins *et al.* [29]. This was facilitated through the GRADE PRO online platform. GRADE outlines four quality categories applicable to a body of evidence: high, moderate, low, and very low. RCTs are initially regarded as high-quality evidence. However, five factors can downgrade the evidence quality: methodological shortcomings, inconsistency of results, indirect evidence, inaccuracy, and publication bias. Conversely, the quality of evidence can be upgraded by three aspects: Magnitude of the effect, a dose-response gradient, and the impact of potential confounding factors, as indicated by Guyatt *et al.* [30] Inter-study heterogeneity was assessed using I^2^ statistics, with values indicating low (0-50%), moderate (50-74%), or high (≥ 75%) heterogeneity, following the interpretation guidelines by Melsen *et al*. [31].

### 2.7 Data Analysis: Systematic Review

A detailed synthesis was conducted to thoroughly outline the methodologies used in each study. Essential elements of each study were comprehensively detailed in narrative and tabular forms. The results from the individual qualitative assessments were determined by examining the Heart Rate Variability (HRV) measurements in resting conditions compared to the values post-exercise, both with and without the influence of CAF. In terms of HRV measurements, the studies clearly defined each metric measured RMSSD refers to the root mean square of successive differences in normal RR intervals; SDNN is the standard deviation of all normal RR intervals within a given time frame, measured in milliseconds (ms). The HF index is the high-frequency segment of power spectral analysis, spanning from 0.15 Hz to 0.44 Hz in both absolute (ms2) and normalized units (n.u.), indicative of vagal activity; LF represents the low-frequency segment, covering 0.04 to 0.15 Hz in both absolute (ms2) and normalized units (n.u.), reflecting both sympathetic and parasympathetic responses. The LF/HF ratio calculates the balance between low and high frequencies; SD1 (derived from poincare plots) quantifies the standard deviation of the immediate variability in heart rate, whereas SD2 (also from poincare plots) measures the standard deviation in long-term RR interval variability. ApEn, or Approximate Entropy, alongside Symbolic Analysis, were employed. In Symbolic Analysis, V0 indicates uniformity in three symbols (dominant sympathetic modulation); V1 shows two consecutive identical symbols with one differing (indicative of both sympathetic and parasympathetic modulation); V2 is when all symbols differ from their predecessors (signifying parasympathetic modulation). To characterize the study populations, data were extracted on the following parameters from each study: author, publication year, participant number, age in years, weight in kilograms, height in centimeters, Body Mass Index (BMI) in kg/m^2^, peak oxygen consumption in mL/kg/min, intensity of exercise, timing of CAF intake, CAF dosage, analysis duration, HRV metrics, and key findings.

### 2.8 Data Analysis: Meta-analysis and Meta-regression

The inclusion of HRV values in the meta-analysis focused on the RMSSD and HF indices (*i.e.* vagal modulation). To justify the use of RMSSD and HF in assessing parasympathetic tone after exercise, it is important to understand their specific characteristics and how they differ from other indices in HRV analysis. RMSSD: This index specifically focuses on short-term variability in the intervals between heartbeats (RR intervals). Unlike other indices like SDNN, which reflect both sympathetic and parasympathetic influences on the heart, RMSSD is more sensitive to changes in parasympathetic activity. This is because rapid fluctuations in RR intervals are predominantly mediated by the parasympathetic nervous system. Therefore, RMSSD is considered one of the best indicators of cardiac parasympathetic modulation, especially useful for post-exercise evaluation when this activity is crucial for recovery. HF: The high-frequency component in HRV spectral analysis is closely related to respiratory variability (sinusoidal breathing) and is predominantly modulated by the parasympathetic nervous system. While indices like LF (low frequency) can be influenced by both sympathetic and parasympathetic activity, HF is more specific to parasympathetic activity. This makes HF a reliable index for assessing the parasympathetic influence on the heart, particularly in post-exercise contexts, where parasympathetic activity is essential for recovery and heart rate reduction. Compared to other indices, RMSSD and HF are thus more specific and sensitive to variations in parasympathetic activity, making them preferable choices for studying the parasympathetic nervous system's response after exercise. Choosing these indices helps to gain a clearer understanding of cardiac autonomic function in response to physical stress and subsequent recovery [32]. CAF intervention effects on each index (RMSSD and HF) were quantified using mean and standard deviation (MSD). MSD was assessed as the percentage difference between experimental CAF and placebo trials, both before (baseline) and after exercise (% = after exercise/before exercise*100). For the meta-analysis, only the initial post-exercise period was considered. Heterogeneity was gauged using the I2 statistic, with a value exceeding 50% indicating substantial heterogeneity between the tests [27]. Standardized mean difference (SMD) values from each RCT were aggregated using a random-effects model (if heterogeneity was significant) or a fixed-effects model (if heterogeneity occurred by chance). Subgroup analysis was conducted based on CAF form (capsule, drinks, and gum) and time of intake (10, 45, and 60 min). To explore the impact of varying CAF doses on vagal reactivation after exercise, meta-regression analyses were employed. This approach served as an alternative to minimize bias associated with different CAF doses. Data organization was done in Microsoft Excel, and all analyses were performed using Comprehensive Meta-Analysis Software 2.2 (Biostat, Englewood, NJ, USA). Statistical significance was denoted by *p <* 0.05.

## 3 RESULTS

**Fig. (1):**
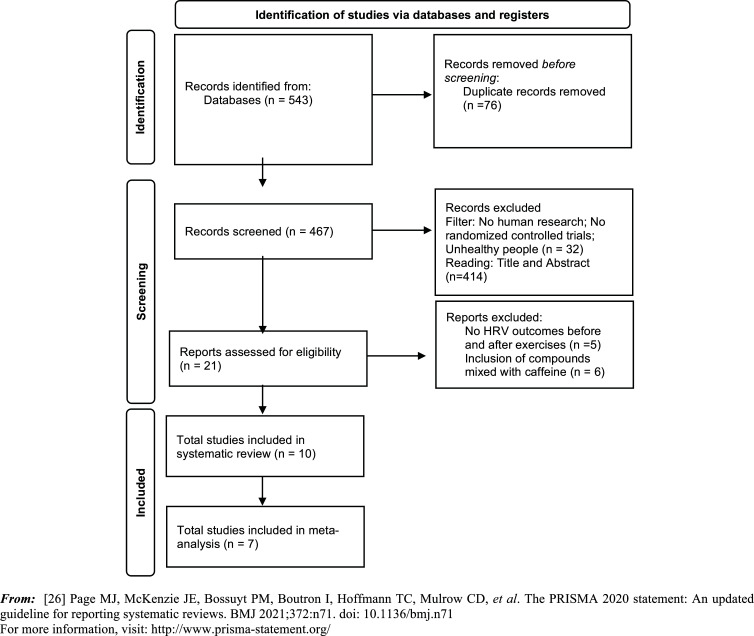
Flowchart prism.

In the process of our comprehensive literature search across four distinct databases, we initially identified a total of 543 studies. Following the removal of 76 duplicate entries, we evaluated 467 articles for their relevance to our research criteria. During this phase, 32 articles were filtered out based on specific exclusion criteria, and an additional 414 articles were dismissed after a preliminary examination of their titles and abstracts. This process left us with 21 articles, which were then subjected to a thorough full-text review. Of these, 11 did not meet our inclusion criteria and were therefore excluded. Consequently, we incorporated 10 studies in our qualitative synthesis for the systematic review. Additionally, 7 of these studies were deemed suitable for inclusion in our quantitative synthesis, forming the basis of our meta-analysis. The details of the research process and selection stages are presented in the PRISMA flow diagram (Fig. **1**).

The figure in Fig. (**2**) illustrates the risk of bias assessment for the chosen studies. In terms of the randomization process, two studies were identified as having a high risk of bias, and two studies were categorized with some concerns, particularly when authors were unclear about the procedure adopted in this aspect. In terms of bias arising from the period and carry-over effects, four studies exhibited some concerns. Regarding deviations from intended interventions, two studies were found to have a high risk of bias, while two studies had some concerns. Missing outcome data were identified in two studies, with one study raising some concerns. In the domain of outcome measurement, two studies were deemed to have a high risk of bias, and two studies raised some concerns. In terms of the selection of reported outcomes, seven studies raised some concerns.

Table **2** presents the level of evidence (determined using the GRADE tool) for the meta-analyzed studies on the form (*i.e.*, capsule, drink, and gum) and time of CAF intake (*i.e.*, 10, 45, and 60 min), based on the indices of vagal response (RMSSD and HF). The evaluations revealed a moderate level of evidence in the studies.

Tables **3** and **4** present the general characteristics of the studies included in the systematic review and meta-analysis.

Seven studies [6, 11, 12, 18-20, 22] were included in a joint analysis of outcomes involving vagal reactivity in time and frequency domain indices (RMSSD and HF), using different forms of CAF intake (capsule, drink, and gum). We applied a fixed effects model and SMD to estimate the effect size (represented by white and black diamonds). No statistically significant difference (*p>*0.05) was found between the conditions (placebo *vs.* caffeine) for both the RMSSD index (heterogeneity I^2^ of 0.00%; Overall: SMD, -0.03; 95% CI, -0.265 to 0.197; *p =* 0.77) and the HF index (heterogeneity I^2^ of 0.00%; Overall: SMD, -0.061; 95% CI, -0.272 to 0.150; *p =* 0.57).

Seven studies [6, 11, 12, 18-20, 22] were included in a joint analysis of outcomes involving vagal reactivity in time and frequency domain indices (RMSSD and HF), using different times of CAF ingestion before exercise (10, 45, and 60 min). We applied a fixed effects model and SMD to estimate the effect size (represented by white and black diamonds). No statistically significant difference (*p>*0.05) was found between the conditions (placebo *vs.* caffeine) for both the RMSSD index (heterogeneity I^2^ of 0.00%; Overall: SMD, -0.03; 95% CI, -0.265 to 0.197; *p =* 0.77) and the HF index (heterogeneity I^2^ of 0.00%; Overall: SMD, -0.061; 95% CI, -0.272 to 0.150; *p =* 0.57).

We conducted a meta-regression analysis using the fixed-effects model, considering the administered CAF doses in the included studies. However, we did not observe any significant association in HRV indices (RMSSD and HF) (*p* > 0.05). These findings indicate that the CAF doses employed in the studies do not have a detrimental effect on vagal reactivity following exercise.

## 4 DISCUSSION

This study aimed to systematically review and conduct a meta-analysis to explore how CAF intake strategies affect HRV indices during post-exercise recovery. The findings from this systematic review and meta-analysis suggest that strategies involving CAF encompassing form, time, and dose of intake do not significantly alter HRV indices during the recovery period following physical exertion, in comparison to a placebo. This conclusion is predicated upon time-domain indices (RMSSD) and frequency (HF) analyses (*i.e.* vagal modulation).

In individuals with athletic training, muscle fatigue manifests as the incapacity to continuously produce muscle strength or power for a determined duration [33]. Under these circumstances, CAF has been recognized as a legitimate ergogenic aid, proven to effectively boost performance across a spectrum of sports, notably those predominantly dependent on muscular power [34] and endurance [7].

The utilization of CAF strategies, including the mode of intake during exercise and the post-exercise recovery period, carries a significant risk of adverse effects such as changes in cardiac autonomic modulation [12, 35, 36]. These disturbances have the potential to contribute to the onset of rhythm disorders and abnormal HRV responses [18]. It is pertinent to emphasize that the effect of CAF on HRV may vary depending on its form of intake. Evidence suggests that CAF in liquid [22] and gum [20] forms may exert a more pronounced influence on reducing parasympathetic activity parameters compared to capsules [17, 36].

**Fig. (3):**
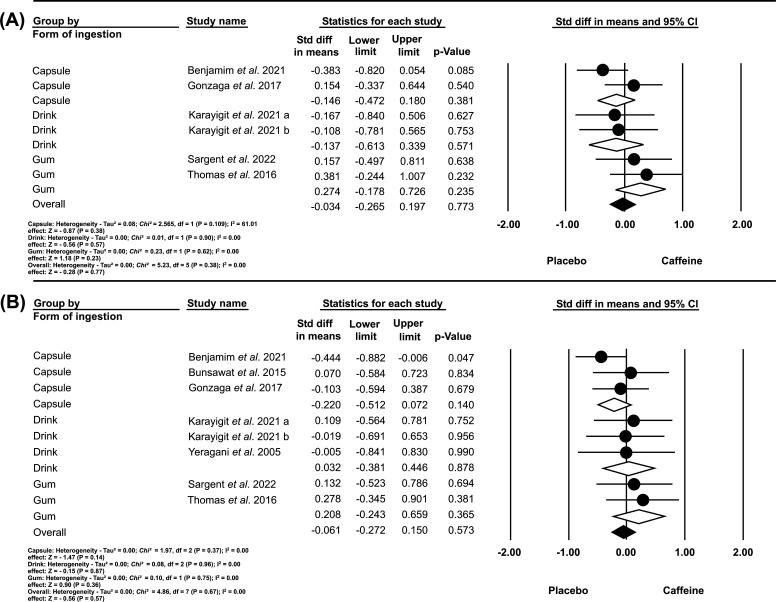
**A**. Effects of the form of caffeine intake before exercise on the recovery of RMSSD indices after exercise. **B**. Effects of the form of caffeine intake before exercise on the recovery of HF indices after exercise.

This phenomenon can be ascribed to the fact that liquid and gum CAF is already in a readily absorbable state by the buccal mucosa, stomach, and small intestine. This attribute is advantageous when the objective is to observe the short-term impacts of CAF consumption. However, these forms could also elevate the risk of adverse effects linked to sudden changes in cardiac autonomic modulation. In contrast, the absorption of CAF encapsulated within capsules is preceded by a dissolution stage, which may introduce additional temporal considerations [37]. Studies [17, 36, 37] using encapsulated forms of CAF might be more suited for investigating long-term effects or effects that manifest after a latency period, owing to the additional dissolution stage in the absorption process. However, this feature might potentially introduce time delays that could complicate the interpretation of results. In contrast, the results of the current study did not observe changes in HRV indices (Fig. **3**).

**Fig. (4):**
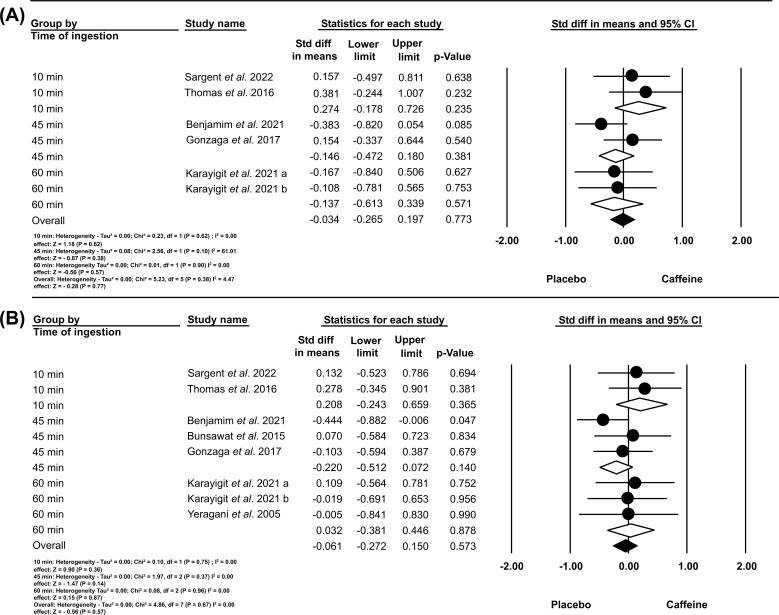
A. Effects of the time of caffeine ingestion before exercise on the recovery of RMSSD indices after exercise. B. Effects of the time of caffeine ingestion before exercise on the recovery of HF indices after exercise.

In line with the guidelines set forth by the International Society of Sports Nutrition (ISSN) [7], there is a strategic emphasis on CAF supplementation. This emphasizes both the timing of ingestion prior to exercise initiation (10, 45, and 60 minutes) and the dosage (ranging from 2.1 to 6.0 mg/kg). This approach aims to sustain elevated caffeine concentrations in the bloodstream during exercise [38]. Considering these strategies (time and dose), there has been a growing interest among researchers to examine their safety, particularly through the estimation of HRV parameters. The safety of CAF intake, as evaluated through HRV parameters, may be significantly affected when intake is less than 60 minutes prior to exercise. Based on the studies by Gonzaga *et al.* [18] and Bunsawat *et al.* [19], the impact of CAF on post-exercise recovery after treadmill endurance exercises is particularly pronounced. Gonzaga *et al.* [18] indicate that CAF consumption can impede parasympathetic recovery following exercise, albeit without markedly affecting the respiratory rate, oxygen saturation, or frequency-domain indices of HRV. Drawing a parallel study by Bunsawat *et al.* [19] provides evidence that CAF intake disrupts autonomic recovery after exercise, predominantly attributed to the amplification of sympathetic nerve activity. This heightened activity potentially compromises baroreflex function during the recovery period, leading to an imbalance in autonomic functionality. Therefore, it might predispose young adults to an elevated risk of arrhythmogenic conditions. In contrast, the results of the current study did not show changes in HRV indices at different times of CAF ingestion before exercise (10, 45, and 60 min) (Fig. **4**).

The optimal timing for CAF consumption may depend on its form of ingestion. As previously noted, specific forms of CAF intake, like caffeinated gums and drinks, might be absorbed more swiftly than CAF consumed in capsule form. This implies that based on the mode of consumption, such as drinks or gums, caffeine could be released and assimilated more rapidly into the bloodstream, potentially amplifying its effects on the autonomic system. Therefore, selecting a mode of CAF intake might heighten the arrhythmogenic risk, especially when it allows bodily absorption.

**Fig. (5):**
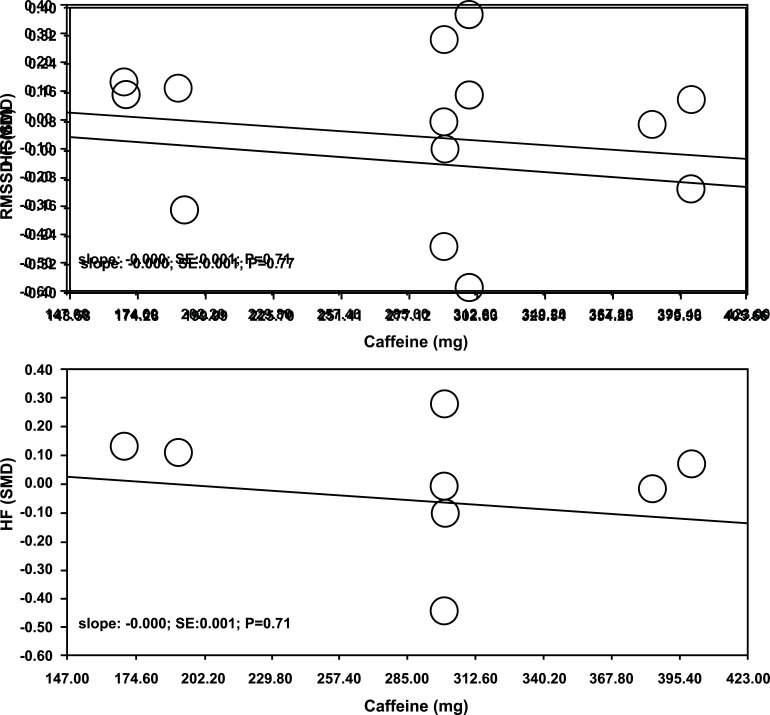
Meta-regression analysis of the association between caffeine doses and effect size (expressed as standardized, average difference [SMD]) of the RMSSD (**A**) and HF (**B**) in vagal reactivity of individuals after exercise.

The studies incorporated into this meta-anlysis used CAF dosages varying between 2.1 and 6.0 mg/kg, levels that are deemed safe according to the Food and Drug Administration (FDA) [39]. The results demonstrated that the selected doses, varying from 192.12 to 400 mg, as used in the referenced studies [6, 11, 12, 18-20, 22] did not show any associations with the delay in vagal reactivity, as observed in the RSSMD and HF parameters (Fig. **5A** and **B**). Despite this, individual sensitivity to the effects of CAF can vary significantly, with regular users possibly developing tolerance due to chronic intake [40, 41]. For individuals not habituated to CAF, it often results in an elevated heart rate and spikes in systolic blood pressure [42]. Yet, these effects typically diminish progressively with ongoing consumption of CAF [43, 44]. For instance, studies by Bunsawat *et al.* [19], Yeragani *et al*. [22], Karayigit *et al.* [6], Sargent *et al.* [11], and Thomas *et al.* [20] employed individuals who consumed daily CAF amounts around 285 mg/day, 62 mg/day, 25 mg/day, 480 mg/day, and 300 mg/day, respectively. This potential physiological adaptation in habituated individuals seems not to influence vagal reactivity response (Fig. **5**).

Evidence has indicated that the number of adenosine (A1) receptors increases following extended CAF administration [45, 46], and most subsequent studies on chronic use have observed an augmentation in these (A1) receptors [47, 48]. This downregulation effect for CAF use is more pronounced in A1 receptors, while the A2A receptors are specifically associated with changes in Gs proteins or adenylate cyclase enzyme [49]. Due to the challenges in quantifying the expression of tissue receptors (muscular, neural, and cardiac) involved in CAF interactions in human models, many researchers have indirectly observed these responses through physiological outcomes such as HRV and heart rate [17, 41].

The research conducted by Thomas *et al.* [20] highlights the importance of recognizing that genetic factors may also play a role in how caffeine impacts HRV recovery. Furthermore, according to the study by Thomas *et al.* [20], it is crucial to consider that the effects of caffeine on HRV recovery may also be influenced by genetic factors. For example, there are significant clinical implications associated with the CYP1A2*1F polymorphism. An analysis of post-exercise recovery data suggests a delay in the vagal response during the CAF trial for those carrying the C allele (slow metabolizer), in contrast to the A/A homozygous (fast metabolizer) individuals. These findings highlight the importance of a more in-depth understanding of caffeine's interaction with HRV, considering the form, time, dose of intake, and individual genetic variations.

The stimulating effect of CAF is well-established, yet various practical aspects pertaining to its effect on post-exercise autonomic recovery require standardization. The present meta-analysis identifies key features that can serve as a roadmap for future research investigating CAF's impact on the autonomic nervous system. Our analysis strongly suggests that CAF intake shows virtually no adverse effects on the cardiovascular system during the post-exercise recovery period. Therefore, it can be confidently incorporated into competitive sports as a potential performance enhancer.

Additionally, in this comprehensive analysis, we explore the complex interaction between caffeine intake and HRV indices, highlighting temporal nuances and differentiated impacts across various contexts. We note that while CAF exerts a pronounced acute effect, HRV demonstrates its influence over extended periods (up to 10 minutes). This temporal distinction is crucial for understanding both specific subgroup analyses and broader assessments, indicating a potentially minimal influence of CAF on HRV. Furthermore, detailed studies have revealed a variety of effects of CAF in different populations and sports modalities [50-56]. From improvements in physical performance and fatigue reduction in female volleyball athletes [57] to increases in power test performance in boxers [58], these findings underscore the potential of caffeine as an effective nutritional strategy to optimize performance and physiological responses during exercise, providing valuable insights for healthcare and sports professionals.

A limitation of our results resides in the demographic specificity of the included studies, as they predominantly investigated the impacts of CAF in a cohort of young, physically active, and ostensibly healthy individuals. This focus significantly narrows the potential for extrapolation of our findings to other population segments. Despite the inability to classify CAF users based on daily consumption by individuals due to data limitations for conducting subgroup analyses, it is essential to note that such constraints do not compromise the integrity or validity of this study's conclusions.

## 5 PRACTICAL APPLICATION

This finding provides practical implications for the field of sports nutrition and athletic training, suggesting that CAF, a widely utilized ergogenic aid, can be strategically incorporated into athletes' dietary and training protocols without compromising post-exercise autonomic recovery (up to 10 minutes). However, it is essential that each athlete's individual response to CAF is carefully monitored and that CAF consumption strategies are individualized for optimal performance improvement. Future research is warranted to elucidate further how various factors, such as genetic polymorphisms, habitual CAF usage, and absorption rates influenced by the form of intake, might interplay with caffeine's effects on HRV recovery and overall exercise performance.

## CONCLUSION

In conclusion, there is moderate certainty evidence suggesting that different CAF intake strategies, encompassing aspects such as form, time, and dose, do not have a significant impact on HRV indices recovery post-exercise (up to 10 minutes). This consistency was predominantly observed among individuals who are either healthy or physically active, as indicated by HRV indices representing parasympathetic cardiac regulation.

## Figures and Tables

**Fig. (2):**
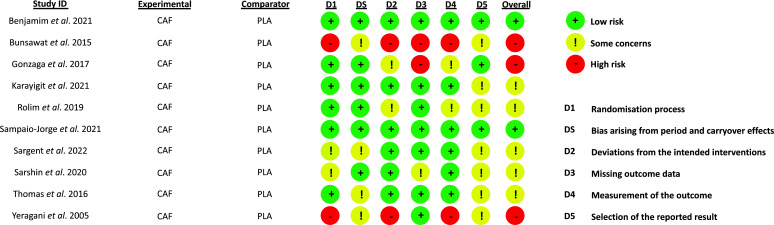
Bias risk analysis judgment by study and bias domains. **Source**: Figure was produced using an Excel tool designed to implement the RoB 2 framework, referenced in our manuscript as [27]. This tool and Microsoft Excel were instrumental in our data organization and analysis, ensuring the originality of all visual contributions.

**Table 1:** Electronic databases used and search strategies. Date: 31/05/2023.

**Database**	**Search Strategy**
PubMed	((“autonomic nervous system”[MeSH Terms] OR (“autonomic”[All Fields] AND “nervous”[All Fields] AND “system”[All Fields]) OR “autonomic nervous system”[All Fields] OR ((“heart rate”[MeSH Terms] OR (“heart”[All Fields] AND “rate”[All Fields]) OR “heart rate”[All Fields]) AND (“variabilities”[All Fields] OR “variability”[All Fields] OR “variable”[All Fields] OR “variable s”[All Fields] OR “variables”[All Fields] OR “variably”[All Fields]))) AND (“caffein”[All Fields] OR “caffeinated”[All Fields] OR “caffeine”[MeSH Terms] OR “caffeine”[All Fields] OR “caffeine s”[All Fields] OR “caffeines”[All Fields] OR “caffeinism”[All Fields]) AND (“exercise”[MeSH Terms] OR “exercise”[All Fields] OR “exercises”[All Fields] OR “exercise therapy”[MeSH Terms] OR (“exercise”[All Fields] AND “therapy”[All Fields]) OR “exercise therapy”[All Fields] OR “exercise s”[All Fields] OR “exercised”[All Fields] OR “exerciser”[All Fields] OR “exercisers”[All Fields] OR “exercising”[All Fields])) AND (clinicaltrial[Filter] OR randomizedcontrolledtrial[Filter])
Web of Science	(((ALL=(Autonomic Nervous System)) OR ALL=(Heart Rate Variability)) AND ALL=(Caffeine)) AND ALL=(Exercise)
Scopus	autonomic AND nervous AND system OR heart AND rate AND variability AND caffeine AND exercise AND (LIMIT-TO (DOCTYPE, “ar”))
Latin American e Literatura Caribenha em Ciências da Saúde (LILACS)	(autonomic nervous system) OR (heart rate variability) AND (caffeine) AND (exercise) AND (type_of_study:(“clinical_trials”) AND la:(“en”)) AND (type_of_study:(“clinical_trials”))

**Table 2:** Level of evidence (GRADE).

**Certainty Assessment**							**No. of Participants**		**Effect**		**Certainty**	**Importance**
**No. of Studies**	**Study Design**	**Risk of Bias**	**Inconsistency**	**Indirectness**	**Imprecision**	**Other Considerations**	**Caffeine**	**Placebo**	**Relative (95% CI)**	**Absolute (95% CI)**		
**Form and time of Ingestion (square root of the mean squared - RMSSD)**												
5	RCTs	Serious	Not serious	Not serious	Not serious	None	128	128	-	Mean -**0.034 Smaller** (-0.265 lower to 0.197 higher)	⨁⨁⨁◯ Moderate	IMPORTANT
**Form and time of Ingestion (high frequency - HF)**												
7	RCTs	Serious	Not serious	Not serious	Not serious	None	157	157	-	Mean -**0.061** Smaller (-0.272 lower to 0.15 higher)	⨁⨁⨁◯ Moderate	IMPORTANT

**Abbreviation:** CI: Confidence Interval.

**Note:** The symbol “⨁⨁⨁◯” signifies that the evidence presented is of moderate quality, based on the GRADE criteria.

**Source:** The categorizations and assessments presented were derived from our analysis, following the GRADE guidelines. These guidelines are publicly accessible through the GRADE Working Group website at [https://gdt.gradepro.org/app/]. The results from this framework were meticulously compiled and analyzed using Microsoft Excel, ensuring the precision and reliability of the data presented. References [29] and [30], cited in conjunction with Table **2**, pertain to the application of the GRADE approach in our analysis, underscoring our methodological rigor and the evidence-based foundation of our conclusions.

**Table 3:** Description of the characteristics of the study population of articles by author and year, sample, age (years), weight (kg), height (cm), BMI (kg/m^2^) (mean SD), exercise, average peak oxygen (ml/kg/min), exercise intensity.

**Author/years**	**Study Design**	**Sample**	**Age (years)**	**Weight (kg)**	**Height (cm)**	**IMC (kg/m^2^)**	**Exercise**	**Average Peak Oxygen (ml/kg/min)**	**Exercise intensity**
Sargent *et al.* (2022) [11]	RCT	18 (9 female, 9 male) healthy, college-aged, physically active adults	22.1 ± 2.6	80.0 ± 10.0	NA	26.9 ± 4.3	Treadmill	Not reported	20 minutes of treadmill walking at a workload corresponding to 60% (calculated) of estimated VO2max.
Sampaio - Jorge *et al.* (2021) [17]	RCT	14 male recreationally-trained cyclists	34.1±4.4	79.1±11.8	178±9	24.6±2.1	Time trial cycling test/ Cycle ergometer	Not reported	Continuous test: the participant was instructed to cover the distance of 16 km in the work intensity as quickly as possible with 50% of the maximum workload capacity
Benjamim *et al.* (2021) [12]	RCT	30 healthy, college-aged males	23.33±3.15	71.14±12.31	1.63±4.51	23.00±2.75	Resistance (Leg press, squat, abductor and extensor chair)	Not reported	The subjects performed strength exercise at 75% of 1 RM
Sarshin *et al.* (2020) [21]	RCT	20 healthy, recreationally active males	24 ± 2	74.70 ± 7.07	178.8 ± 4.64	Not calculated	Cycle ergometer	Not reported	Each test began with a 4-min standardized warm-up against a fixed load of 1 kilopond and three separate 2 s sprints were performed against a load of 0.075 kp∙kg^-1^ of body mass were interspersed with 45-s of active recovery
Karayigit *et al.* (2021) [6]	RCT	17 (rugby: 10, handball: 4, and soccer: 3) healthy, non-smoking, resistance trained female team sport athlete	23 ± 2	64 ± 4	168 ± 3	Not calculated	Resistance (Squat and Bench press)	Not reported	Three sets of repetitions to failure with the load of 40% of 1 RM for the squat and bench press.
Rolim *et al.* (2019) [35]	RCT	21 healthy physically active young men	22.3 ± 2.9	Not reported	Not reported	25.2 ± 2.7	Treadmill	Not reported	Three minutes of warm-up at 3 km/h and 2.5% of slope. After this period, increments of 1 km/h were made each minute, with no change of inclination, until volunteers reached 85% of maximal heart rate (HRmax)
Gonzaga *et al.* (2017) [18]	RCT	32 healthy young male volunteers	23.59±3.45	78.87±12.14	1.79±7.14	24.40±2.82	Treadmill	44.00±12.25	Volunteers exercised on the treadmill at a speed of 5 km/h and slope of 1% in the first 5 minutes for warm up, followed by 25 minutes with work load equivalent to 60% of the VO2peak HR
Thomas *et al.* (2016) [20]	RCT	20 apparently healthy, untrained adults (13 males and 7 females)	25.5 ± 3.5	Not reported	Not reported	26.2 ± 4.6	Cycle ergometer	32.2 ± 6.4	Cycled for 15 min at a constant workload set at 75% of their VO2-peak.
Bunsawat *et al.* (2015) [19]	RCT	18 healthy individuals	26 ±1.0	Not reported	Not reported	23.9 ± 0.8	Treadmill	Not reported	The participants underwent a treadmill exercise test to assess maximum oxygen consumption (VO2max), followed by 2 minutes active recovery at a speed of 3.5km/h and grade of 0%.
Yeragani *et al.* (2005) [22]	RCT	11 (6 males and 5 females) heathy subjects	Male: 29.0 ± 7.0 Female: 23 ± 7.0	Not reported	Not reported	Not calculated	Cycle ergometer	Not reported	The graded exercise consisted of stationary ergometer cycling for 3-min stages progressing at 25-W increments. Thus, the intensity of the exercise started out relatively easy, and every 3 min was increased until participants reached volitional fatigue.

**Table 4:** Description of the selected articles by author and year, time ingestion CAF, CAF dose, placebo, analysis time, HRV index and main conclusions.

**Author/ Years**	**Ingestion CAF (Before Exercise)**	**CAF Dose**	**Placebo**	**Analysis Time**	**HRV Index**	**Main Conclusions**
Sargent *et al.* (2022) [11]	10 min	(2.1 mg/kg) 170 mg	Gum	Post-exercise HRV was assessed between 0 – 10 minutes (post 1), 10 – 20 minutes (post 2), and 20 – 30 minutes (post 3)	SDNN, RMSSD, LF, HF, LF/HF and SD1	CAF did not delay the recovery of HRV indices reflective of parasympathetic nervous system activity following an acute bout of moderate exercise
Sampaio - Jorge *et al.* (2021) [17]	60 min	(6 mg/kg) 479.6 mg	250 mg magnesium silicate (capsule)	Successive RR intervals were acquired for 5 min while supine (REST) and along the TT-test. 3 times, before the ingestion of the capsule (PRE suppl), 60 min afer supplementation (PRE exerc), and 10 min after the TT-test (POST)	Symbolic analysis: (I) patterns with no variation (V0; all three symbols were equal); (II) patterns with one variation (V1; two consequent symbols were equal, and the remaining symbol was diferent); and (III) patterns with two unlike or like variations (V2; all the symbols were diferent from the previous ones)	Acute CAF intake increased performance (time-trial) and demonstrated a relevant cardioprotective efect, through increased vagal tone
Benjamim *et al.* (2021) [12]	45 min	(4.2 mg/kg) 300 mg	Capsule (starch)	0-5, 5-10, 10-15, 15-20, 20,25 and 25-30 min post exercise	SDNN, RMSSD, RRTri, TINN, SD1, SD2, LF and HF (ms2)	CAF delayed HR and HRV recovery following strength exercise
Sarshin *et al.* (2020) [21]	45 min	(3 mg/kg) 224.1 mg and (6 mg/kg) 448.2 mg	Capsule (dextrose)	0-5 and 30-35 min postexercise	SDNN and RMSSD	CAF improved HRV recovery post-exercise.
Karayigit *et al.* (2021) [6]	60 min	(3 mg/kg) 192.12 mg and (6 mg/kg) 384.24 mg	Decaffeinated coffee	Immediately post-latest exercise (<1 min)	SDNN, RMSSD, TP, HF (ms2) LF (ms2), LF/HF, HF (n.u.) and LF (n.u.)	CAF protocols did not demonstrate changes in HRV recovery between interventions.
Rolim *et al.* (2019) [35]	60 min	(3 mg/kg)	Capsule (not specified)	60 and 300 s post-exercise, with the treadmill velocity reduced to 2.4 km/h.	SD1 and SD2	CAF accelerated parasympathetic reactivation in the submaximal exercise test in the first 3 min.
Gonzaga *et al.* (2017) [18]	45 min	(3.80 mg/kg) 300 mg	Capsule (starch)	0-5, 5-10, 10-15, 25-30, 35-40, 45-50 and 55-60 min post-exercise	RMSSD, SD1, SDNN, HF, LF and LF/HF	Intake CAF before exercise delayed the parasympathetic recovery following moderate aerobic exercise.
Thomas *et al.* (2016) [20]	10 min	300 mg	Gum	0-5 and 5-10 after exercise	SDNN, RMSSD, LF (nu), HF (nu), LF/HF, SD2 (ms), ApEn	CAF did not impaired HRV indexes recovery after exercise.
Bunsawat *et al.* (2015) [19]	45 min	(5.06 mg/kg) 400 mg	Pills (not specified)	5, 15 and 30 min postexercise	LF/HF	CAF protocol delayed vagal reactivation after exercise.
Yeragani *et al.* (2005) [22]	60 min	(5 mg/kg) 300 mg	Cola drink	0-5 min after exercise	LF (ms2), HF (ms2), LF/HF, ApEn	CAF delayed HRV recovery after exercise.

## Data Availability

The authors confirm that the data supporting the findings of this research are available within the article.

## LIST OF ABBREVIATIONS

CAF: Caffeine
HRV: Heart Rate Variability
RMSSD: Root Mean Square of Succes Sive Difference
SMD: Standardized Mean Difference
HF: High Frequency

## STANDARDS OF REPORTING

PRISMA guidelines and methodology were followed.

PRISMA checklist is available on the publisher’s website.

## SUPPLEMENTARY MATERIAL

PRISMA checklist is available as supplementary material on the publisher’s website along with the published article. Supplementary material is available on the publisher’s website along with the published article.
